# Changes in launch delay and availability of pharmaceuticals in 30 European markets over the past two decades

**DOI:** 10.1186/s12913-022-08866-7

**Published:** 2022-11-30

**Authors:** Melanie Büssgen, Tom Stargardt

**Affiliations:** Hamburg Center for Health Economics, University of Hamburg, Hamburg, Germany

**Keywords:** Launch delay, Availability, Pharmaceuticals, Europe, Health Policy, EU

## Abstract

**Background:**

The timing of the launch of a new drug is an important factor that determines access for patients. We evaluated patient access to pharmaceuticals in 30 European markets over the past two decades.

**Methods:**

Launch dates were extracted from the IQVIA (formerly IMS) database for 30 European countries for all pharmaceuticals launched internationally between 2000 and 2017. We defined launch delay as the difference between the first international launch date and the corresponding national launch date, and calculated these for each country in our sample over time. Additionally, we ranked countries according to their launch delays and looked at changes in the ranking order over time. Lastly, we determined the availability of new pharmaceuticals in each country, calculating this as the percentage of these pharmaceuticals that were available in each country during a pre-specified interval.

**Results:**

There was a clear trend towards a decrease in launch delays across all countries from 2000 (37.2 months) to 2017 (11.8 months). Over the entire observation period, the three fastest launching countries were the Netherlands, Sweden, and Germany, whereas the three slowest were Bosnia-Herzegovina, Serbia, and Turkey. Germany had the highest availability of new pharmaceuticals with 85.7%, followed by the United Kingdom (83.1%) and Norway (82.9%). Countries with the lowest availability of pharmaceuticals were Bosnia-Herzegovina, Serbia, and Latvia. Gross domestic product per capita was negatively correlated with launch delay (-0.67, *p* < 0.000) and positively correlated with the availability of pharmaceuticals (+ 0.19, *p* < 0.000).

**Conclusion:**

Launch delay and the availability of pharmaceuticals varied substantially across all 30 European countries. Using countries with above-average availability and below-average launch delays as a benchmark, stakeholders may discuss or modify current pharmaceutical policy, if needed, to improve access to pharmaceutical care.

**Supplementary Information:**

The online version contains supplementary material available at 10.1186/s12913-022-08866-7.

## Background

Health systems in Europe are facing a range of challenges, including how best to care for an ageing population and an increasing number of people diagnosed each year with cancer [1] and chronic illnesses [2]. At the same time, the cost of new pharmaceuticals and other technologies continues to rise. In fact, spending for pharmaceutical care in Europe in 2020 was higher than ever before [3]. To balance the trade-off between costs and access to safe, quality health care, including essential medicines, European countries have strengthened reimbursement schemes for pharmaceuticals over the past two decades, thereby limiting growth in pharmaceutical prices. Regulating prices, however, comes at the expense of making markets less attractive to pharmaceutical companies [4], with potentially negative ramifications for patient access [5].

Sufficient access to pharmaceuticals is crucial to staying healthy or achieving health [6] and has been enshrined as part of the right to health in international law since 1946. It is widely recognised that access to modern medical treatments has contributed immensely to improving patients’ quality of life [7], and that a lack of access to these can compromise health outcomes [8]. Some well-evidenced barriers to accessing pharmaceuticals include data exclusivity [9, 10], costs [11–13], a lack of generic brands [13, 14], and patent protection and exclusivity [9, 15, 16]. Prior research also suggests that delays in the launch of pharmaceuticals may result in some patients not being able to access new treatments in a timely fashion. This can lead to loss of life years and lower quality of life [17], particularly if patients cannot tolerate the standard treatment options or if these are contraindicated or substantially less effective than the new pharmaceuticals.

Delays in the launch of new pharmaceuticals in Europe, and in particular how these delays have developed over time have been the subject of little research to date. We aim to fill this gap in the literature by analysing the dynamics of launch delays and availability of pharmaceuticals in 30 European countries over two decades. Results are of interest to supply chain facilities (manufacturers, distributors, wholesalers) and can also inform discussions about pharmaceutical policy and help stakeholders and policymakers identify where existing regulations and laws may require improvement.

## Methods

### Data extraction

We extracted the first international launch date and national launch dates of prescription drugs from the IQVIA (formerly IMS Health) Database for the following 30 European countries: Austria, Belgium, Bosnia-Herzegovina, Bulgaria, Croatia, Czechia, Estonia, Finland, France, Germany, Greece, Hungary, Ireland, Italy, Latvia, Lithuania, Luxembourg, Netherlands, Norway, Poland, Portugal, Romania, Serbia, Slovakia, Slovenia, Spain, Sweden, Switzerland, Turkey, and the United Kingdom (UK). The dataset has been used for distantly related research before [5, 18, 19].

We restricted our sample to prescription drugs that were launched internationally between 2000 and 2017 in the retail or hospital market. Our follow-up period for national launch dates was from 2018 until the second calendar quarter of 2020. We distinguished between four time intervals to examine how the values of our indicators changed over time: 2000–2004, 2005–2009, 2010–2014, and 2015–2017.

To avoid confounding (a) by pharmaceuticals launched very late in some countries after already having been taken from the market in other countries, and (b) peculiarities in being counted as a (prescription) drug, two researchers independently reviewed all pharmaceuticals that were launched in fewer than five countries to verify that the international launch date given in the database was correct. Furthermore, we excluded vaccines from the analyses because these were marked as newly launched in the database in cases where a booster of an existing vaccine had been launched.

### Statistical analysis

We operationalized access to newly discovered pharmaceuticals in three ways: First, we calculated the length of time between the first international launch date of each drug and its corresponding national launch date in each country and defined this as the launch delay. Second, we ranked each country in our sample according to the average launch delay across all new pharmaceuticals in that country, and we analysed changes in the ranking order of countries by year and across the four time intervals defined above. Third, we measured differences in the overall availability of new pharmaceuticals across the countries in our sample over time. We did so by determining the number of pharmaceuticals launched internationally during each of the four time intervals and calculating the percentage of these pharmaceuticals that were available in each country during that interval. For this last indicator, we excluded Estonia, Greece, and Luxembourg from our analyses because the IQVIA collects data only for the retail market in these countries.

To investigate whether pharmaceuticals classified as essential medicines by WHO (EMLs) [20] follow similar trends in launch delay and whether availability of pharmaceuticals differs compared to all other drugs, we analysed launches of EMLs in a subgroup analysis. In our dataset we found 46 launches to be part of the 482 medicines listed on EMLs (9.5%).

### Subgroup analyses

Furthermore, we sought to understand whether our measure of launch delay and the availability of newly discovered pharmaceuticals differed between (a) drugs that covered a potentially urgent need (i.e., first-in-class innovations) and (b) drugs that were similar to, and without substantial therapeutic advantage over existing products (i.e., so-called me-too products). To do so, we examined pharmaceuticals to treat diabetes (DPP-4 inhibitors) and psoriasis (monoclonal antibodies) more specifically in a subgroup analysis. We differentiated between sitagliptin and all other DPP-4 inhibitors, and adalimumab and all other monoclonal antibodies for psoriasis. We chose diabetes and psoriasis as therapeutic areas, as data was most robust for these. I.e., in diabetes and psoriasis, we observed most launches in our time period.

### Correlation analysis

Furthermore, we extracted annual data on the gross domestic product (GDP) per capita of all 30 countries in order to correlate these with the launch delay and availability of pharmaceuticals [21]. Because the investigated variables were not normally distributed (all *p*-values were highly significant using the Shapiro-Wilk test: launch delay (*p* < 0.000), availability of pharmaceuticals (*p* < 0.000), GDP (*p* < 0.000)), we employed correlation analysis according to Spearman (for launch delay) and a point biserial correlation (for availability).

All analyses were performed using Stata SE 16.

## Results

### Sample descriptives

Our final sample included 492 different molecules, of which 124 were launched between 2000 and 2004, 120 between 2005 and 2009, 158 between 2010 and 2014, and 90 between 2015 and 2017. Across the whole sample of 30 countries, there was a mean launch delay of 35.96 months in the first period (2000–2004), 27.52 months in the second period (2005–2010), 22.69 months in the third period (2010–2014), and 14.80 months in the fourth period (2015–2017). When looking at the mean launch delay across all pharmaceuticals in the five biggest European pharmaceutical markets, France was ranked as the country with the 12th shortest launch delay, Germany with the 5th shortest launch delay, Italy with the 11th, Spain with the 6th, and the UK with the 3rd in 2000. In 2017, however, France was ranked as the country with the 13th shortest launch delay, Germany with the 3rd shortest launch delay, Italy with the 14th, Spain with the 18th, and the UK with the 4th. Lastly, of the pharmaceuticals that were launched internationally during the four periods of our analysis, 69.86% were available in our sample of applicable countries in the first period (2000–2004), 65.21% in the second period (2005–2009), 66.50% in the third period (2010–2014), and 52.63% in the fourth period (2015–2017).

### Launch delay

Generally, we saw a dynamic towards a decrease in launch delay across all investigated countries in our sample, with this delay falling from 37.2 months in 2000 to 11.8 months in 2017. From the first period (2000–2004) to the second period (2005–2009), the mean launch delay decreased by 8.9 months. From the second period (2005–2009) to the third period (2010–2014), the launch delay decreased on average by another 4.9 months. From the third period (2010–2014) to the fourth period (2015–2017), the launch delay decreased on average by 7.7 months. For example, for the five biggest European pharmaceutical markets, the launch delay decreased (from the first to the third period) from 33.0 to 24.1 months in France, from 19.4 to 11.5 months in Germany, from 30.7 to 20.7 months in Italy, from 26.4 to 23.5 months in Spain, and from 20.4 to 10.9 months in the UK. For the five smallest European pharmaceutical markets, the launch delay decreased from 99.5 to 45.2 months in Bosnia-Herzegovina, from 71.9 to 45.5 months in Serbia, from 61.3 to 33.7 months in Croatia, from 44.8 to 24.4 months in Slovenia, and from 51.9 to 30.9 months in Romania. The largest decrease in launch delay, of 36.46 months, was seen for Bosnia-Herzegovina from the first period to the second period. However, this decrease should be interpreted with caution given the restricted length of follow-up.

Changes in the ranking order of countries by launch delay occurred over the entire observation period. For example, for the five biggest European pharmaceutical markets, we found that France went from rank 12.8 in 2000–2004 to rank 17.0 in 2015–2017, Germany went from rank 4.6 to rank 3.8, Italy from rank 13.4 to rank 14.4, Spain from rank 9.0 to rank 18.3, and the UK from rank 5.0 to rank 3.4, with smaller ranks indicating shorter mean launch delays. The three countries that were able to improve their rank (i.e., reduce the launch delay) the most from 2000 to 2004 to 2015–2017 were Latvia (− 9.15 ranks), Slovenia (-7 ranks), and Luxembourg (-6.5 ranks). The 3 countries whose ranks worsened the most (i.e., due to an increase in launch delays) were Ireland (+ 10.65 ranks), Spain (+ 9.25 ranks), and Switzerland (+ 5.2 ranks).

For EML-drugs launch delay seems to be roughly the same compared to all new pharmaceuticals in 2004–2010. Thereafter (2011–2014) launch delay was shorter for EML-drugs compared to all other pharmaceuticals. Data for 2015–2017 could not be analysed as only a single drug listed on EML was launched (see appendix, Table S2).

For our subgroup analysis looking at diabetes and psoriasis, we observed similar launch delays for first-in-class innovations and me-too products. The mean launch delay across all 30 European countries was 24.05 months for first-in-class innovations and 25.10 months for me-too products.

Correlation analyses using Spearman’s rank correlation coefficient showed that launch delay was negatively correlated with a country’s GDP per capita (-0.67, *p* < 0.000), i.e., the higher the GDP, the shorter the launch delay (see Table S1, Appendix).

**Fig. 1:**
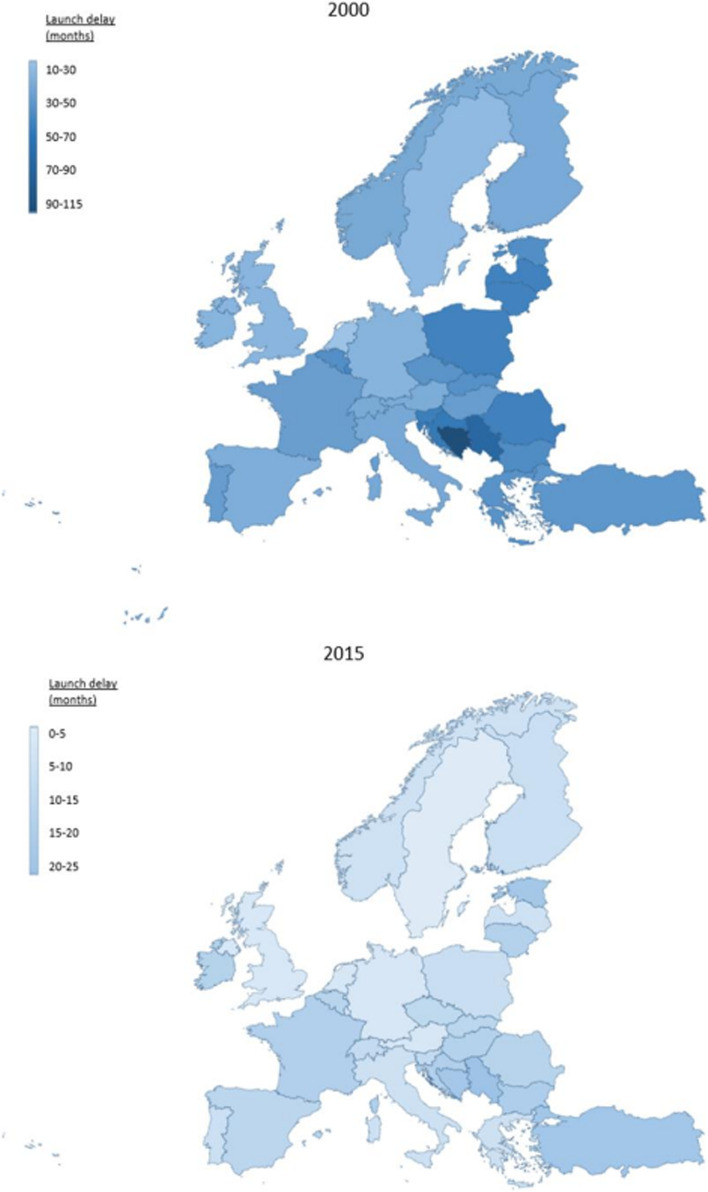
Launch delay in months in 30 European markets 2000 vs. 2015. Dark blue marks countries with short launch delays, and light blue marks countries with long launch delays

**Fig. 2:**
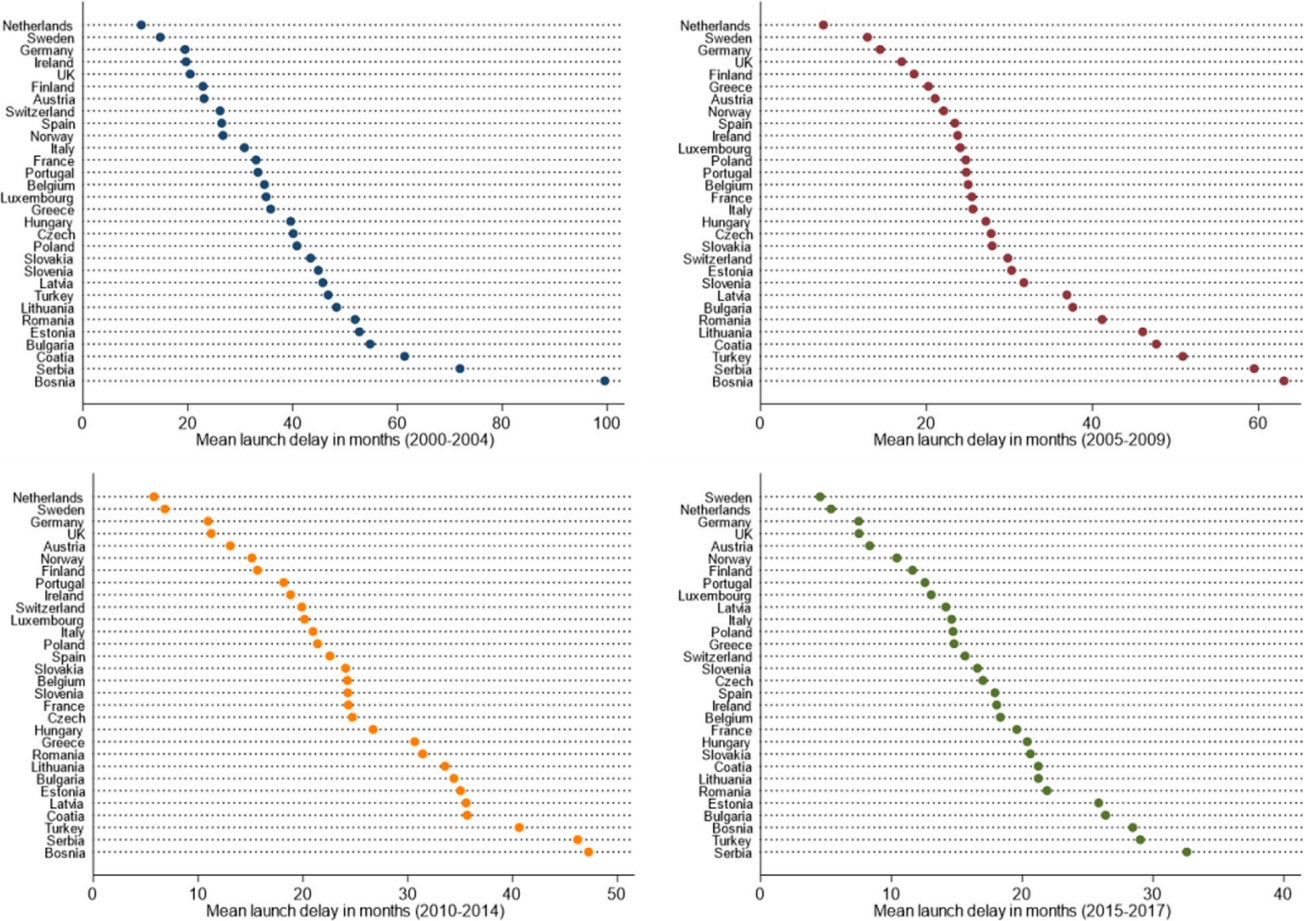
Ranking order of launch delays across 30 European countries in all four time intervals

Figure 1 gives an overview of launch delays in 30 European markets in 2000 versus 2015. Table 1 reports the descriptive statistics for the yearly launch delay over time. Changes in the ranking order of launch delays over time can be seen in Fig. 2.

**Table 1:** Average launch delay across all 30 investigated countries from 2000-2017 in months

Country	Launch delay								
	*2000* *(months)*	*2001* *(months)*	*2002* *(months)*	*2003* *(months)*	*2004* *(months)*	*2005* *(months)*	*2006* *(months)*	*2007* *(months)*	*2008* *(months)*
Austria	24.8	28.7	21.8	20.3	16.0	31.3	23.3	21.0	18.4
Belgium	43.6	36.4	30.1	31.0	31.5	31.0	25.4	22.5	23.5
Bosnia-Herz.	114.5	106.1	95.7	90.4	85.4	75.9	81.2	52.8	72.4
Bulgaria	45.6	73.7	61.7	48.7	29.9	44.6	44.2	38.1	34.0
Croatia	58.6	74.8	59.2	59.9	50.2	44.5	54.1	45.6	46.3
Czechia	40.9	48.7	38.2	40.7	29.6	43.3	18.5	26.6	25.4
Estonia	44.2	76.5	41.8	65.7	31.9	35.5	29.3	20.6	34.1
Finland	25.9	28.8	22.1	14.1	17.6	32.7	19.4	19.2	12.2
France	33.8	41.3	32.6	29.3	21.6	28.3	28.3	22.1	29.0
Germany	19.3	24.7	18.7	21.7	10.1	27.1	13.5	12.7	12.9
Greece	41.3	43.8	31.4	36.8	24.3	25.8	21.3	10.8	29.9
Hungary	34.2	54.2	45.2	29.2	23.9	27.9	34.9	22.2	22.5
Ireland	18.8	31.1	14.2	19.6	12.8	27.6	30.1	18.6	25.7
Italy	26.6	34.1	33.7	30.2	26.0	36.4	26.4	24.8	22.6
Latvia	52.2	56.3	39.6	50.1	29.0	45.7	36.6	26.8	39.4
Lithuania	52.4	55.7	47.4	53.3	25.8	48.4	49.0	46.2	37.0
Luxembourg	49.0	38.1	21.9	34.6	31.5	39.4	10.5	21.7	26.7
Netherlands	9.0	17.5	8.7	12.9	5.8	14.2	5.9	1.4	6.0
Norway	26.2	35.4	27.6	18.4	17.2	29.5	23.4	27.3	13.3
Poland	52.1	48.5	37.3	44.1	19.2	31.1	29.6	16.4	29.3
Portugal	34.4	37.4	35.2	30.5	24.7	35.1	31.3	19.7	24.0
Romania	53.5	67.5	46.9	53.6	34.1	37.5	37.9	38.4	49.4
Serbia	78.9	72.4	72.1	78.5	56.2	64.2	71.0	46.7	59.5
Slovakia	42.7	57.9	39.1	40.7	30.2	38.8	19.5	22.0	34.1
Slovenia	54.4	57.7	42.3	40.4	23.9	38.4	36.7	27.6	26.3
Spain	23.5	31.4	28.5	22.7	22.6	30.9	21.6	20.7	25.5
Sweden	15.0	21.1	14.5	9.8	7.4	18.6	16.8	9.4	15.1
Switzerland	25.6	28.6	29.5	28.6	15.3	38.4	24.6	27.4	33.4
Turkey	39.0	57.1	42.9	55.5	37.9	40.5	48.8	47.6	55.3
UK	18.2	28.6	16.5	22.9	13.1	27.0	18.9	15.3	16.4

Country	Launch delay								
	*2009* *(months)*	*2010* *(months)*	*2011* *(months)*	*2012* *(months)*	*2013* *(months)*	*2014* *(months)*	*2015* *(months)*	*2016* *(months)*	*2017* *(months)*
Austria	13.0	27.7	11.8	15.1	8.7	9.3	8.4	8.9	7.8
Belgium	23.9	37.1	24.7	26.3	20.7	19.3	22.1	16.9	15.5
Bosnia-Herz.	45.2	55.6	61.5	49.6	48.4	24.1	31.9	39.9	23.0
Bulgaria	28.8	26.8	29.0	45.2	35.0	31.9	27.0	26.4	25.3
Croatia	45.2	48.9	38.9	40.9	26.6	30.9	25.6	19.0	18.6
Czechia	28.9	25.7	25.2	32.4	21.5	20.8	19.3	16.4	14.7
Estonia	29.4	50.3	41.3	35.4	30.6	25.2	31.9	20.8	19.9
Finland	10.5	18.5	18.6	18.2	11.8	13.3	14.5	13.0	7.7
France	21.1	26.3	25.4	26.5	25.6	20.8	25.7	18.6	13.3
Germany	7.8	6.7	17.6	12.5	9.5	8.1	8.4	7.9	6.3
Greece	16.9	27.8	39.9	32.3	22.3	28.6	12.7	17.0	19.0
Hungary	26.5	38.2	29.5	32.5	16.3	24.2	24.2	21.3	16.3
Ireland	18.8	15.9	20.4	23.9	16.8	16.2	23.7	17.6	12.9
Italy	19.6	22.1	22.8	24.6	17.2	19.3	13.2	17.1	13.4
Latvia	36.2	40.3	48.5	44.6	23.8	23.1	12.4	17.6	13.2
Lithuania	47.7	35.1	40.0	39.4	29.8	27.2	24.5	21.8	17.0
Luxembourg	20.0	28.5	18.5	24.0	17.1	12.1	9.0	16.6	13.7
Netherlands	9.9	9.1	9.4	4.4	2.5	4.4	8.1	4.8	2.1
Norway	16.7	19.4	19.8	14.6	14.0	10.5	12.3	10.9	7.9
Poland	19.9	34.0	19.8	23.9	16.9	15.8	15.2	15.7	13.3
Portugal	14.0	19.1	20.2	24.7	15.0	13.6	13.9	14.3	9.7
Romania	42.8	34.4	29.4	32.4	36.3	27.1	22.6	18.8	26.4
Serbia	57.0	49.3	51.0	47.0	47.7	39.1	35.5	33.2	25.4
Slovakia	28.1	19.1	27.7	25.6	20.3	25.8	21.5	21.6	18.0
Slovenia	30.8	23.6	25.7	30.3	21.5	21.7	17.0	17.8	15.1
Spain	19.8	16.3	24.4	30.1	21.0	19.8	21.0	17.2	15.1
Sweden	6.8	5.9	9.1	10.6	3.2	5.9	5.4	4.4	3.8
Switzerland	26.8	27.6	23.3	15.6	19.8	17.2	17.1	18.3	11.2
Turkey	58.7	45.0	52.5	42.6	39.3	26.7	32.6	34.3	19.4
UK	9.4	13.8	12.5	11.5	11.0	9.3	7.5	7.7	7.4

### Availability of new pharmaceuticals

In general, the availability of pharmaceuticals that had been newly launched internationally varied significantly across the countries in our sample. The availability of such pharmaceuticals was quite stable from 2000 to 2014, but decreased afterwards, potentially due to the short length of follow-up time which results in less time to observe a launch. For the five biggest pharmaceutical markets in the EU, we found that the availability of new pharmaceuticals decreased from 72.6% in 2000–2004 to 61.1% in 2015–2017 in France, increased from 85.5 to 91.1% in Germany, decreased from 81.5 to 74.4% in Italy, decreased from 82.3 to 70.0% in Spain, and increased from 82.3 to 83.3% in the UK.

Looking at mean values across the whole period from 2000 to 2017, Germany had the highest availability of new pharmaceuticals (85.7%), followed by the UK (83.1%) and Norway (82.9%). The three countries with the lowest availability of new pharmaceuticals over the same period were Bosnia-Herzegovina (24.8%), Serbia (36.4%), and Latvia (43.5%). The three countries in which the availability of new pharmaceuticals decreased the most during this period were Turkey (-16.8% points from 2000 to 2004 to 2010–2014), the Netherlands (-14.0% points), and Bosnia-Herzegovina (-12.0% points).

Availability was generally higher for EML-drugs compared to all other drugs. (see Table 2). For our subgroup indications diabetes and psoriasis, we saw that the availability for first-in-class innovations vs. me-too products differed substantially. The average availability across all 30 European countries was 98.14% for first-in-class innovations and 79.16% for me-too products. Compared to the availability observed across all products (64.49%), availability for the products in our subgroup analysis was quite high. This also implies that at least one DPP-4 inhibitors and at least one monoclonal antibody for psoriasis were available in all markets.

The point biserial correlation analyses showed that GDP was positively correlated with the availability of pharmaceuticals – that is, the higher the GDP, the higher the availability of pharmaceuticals (+ 0.19, *p* < 0.000) (see Table S1, Appendix).

Table 2 reports the descriptive statistics for the availability of new pharmaceuticals over time.

**Table 2:** Availability of new pharmaceuticals +EMLs across 27 investigated countries in four time intervals from 2000-2017

**Country**	**Availability of pharmaceuticals**							
	**2000-2004**		**2005-2010**		**2011-2014**		**2015-2017**	
	**All (%)**	**EMLs (%)**	**All (%)**	**EMLs (%)**	**All (%)**	**EMLs (%)**	**All (%)**	**left out due to low N of EMLs in this period**
Austria	80.6	95.0	78.3	90.9	81.0	92.3	83.3	
Belgium	77.4	90.0	69.2	90.9	73.4	92.3	65.6	
Bosnia	37.9	65.0	21.7	45.5	25.9	53.8	8.9	
Bulgaria	57.3	80.0	55.0	81.8	57.6	76.9	31.1	
Croatia	55.6	75.0	55.0	81.8	60.1	53.8	41.1	
Czech Republic	74.2	90.0	65.0	90.9	75.9	76.9	54.4	
Finland	78.2	90.0	70.8	81.8	75.3	84.6	71.1	
France	72.6	90.0	75.0	81.8	72.2	92.3	61.1	
Germany	85.5	95.0	83.3	90.9	84.8	100.0	91.1	
Hungary	69.4	85.0	63.3	100.0	67.7	84.6	47.8	
Ireland	71.8	90.0	68.3	90.9	68.4	92.3	46.7	
Italy	81.5	95.0	78.3	90.9	84.2	100.0	74.4	
Latvia	56.5	80.0	44.2	72.7	46.8	61.5	18.9	
Lithuania	60.5	85.0	56.7	90.9	55.1	92.3	36.7	
Netherlands	62.1	75.0	52.5	54.5	48.1	61.5	40.0	
Norway	83.9	90.0	80.0	81.8	86.7	92.3	78.9	
Poland	65.3	90.0	69.2	90.9	63.9	69.2	41.1	
Portugal	84.7	100.0	70.0	90.9	72.8	92.3	63.3	
Romania	63.7	80.0	58.3	81.8	53.8	76.9	17.8	
Serbia	46.8	65.0	37.5	63.6	37.3	69.2	18.9	
Slovakia	76.6	90.0	75.0	90.9	72.8	76.9	48.9	
Slovenia	65.3	80.0	65.8	90.9	69.0	69.2	57.8	
Spain	82.3	95.0	83.3	90.9	79.1	92.3	70.0	
Sweden	76.6	90.0	78.3	90.9	79.1	84.6	76.7	
Switzerland	71.8	85.0	70.8	100.0	70.3	76.9	66.7	
Turkey	66.1	75.0	54.2	100.0	49.4	76.9	25.6	
UK	82.3	95.0	81.7	90.9	84.8	100.0	83.3	

## Discussion

In this paper, we examined access to new pharmaceuticals in 30 European markets, placing a special focus on [1] the delay in the launch of new pharmaceuticals in each country compared to the international launch dates and [2] the availability of new pharmaceuticals in each country. In general, we found that the launch delay varied widely across countries and decreased substantially from 2000 to 2017. Furthermore, there were very substantial variations in the availability of pharmaceuticals across our sample, implying that countries with lower expected prices or smaller expected market size have fewer launches and longer launch delays.

Large variation exists among the 30 European countries in our sample, which is perhaps unsurprising given the heterogeneity in how they fund pharmaceutical care. Countries in the geographic east (especially the Baltic countries and those in southeastern Europe) had a longer launch delay and lower availability of pharmaceuticals than, for example, those in the west. It was also notable that the Scandinavian countries and the “Big 5” had rather short launch delays and a high availability of pharmaceuticals.

The substantial decrease in launch delays in Europe over the past two decades can be attributed to globalisation and bigger capacities in pharmaceutical companies to launch their products on a larger scale [22, 23]. Indeed, it has become easier for pharmaceutical companies to operate globally. With different locations worldwide, and through digitalisation and automation [24], economies of scale (mergers and acquisitions) [25], and faster transport routes improving logistics [26], it is now possible to launch products more quickly and often simultaneously in many different countries. Also, increased competition drives companies to launch their products as quickly as possible [27, 28].

This being said, because each country in Europe has a different health system with different pricing and reimbursement schemes, the large variations we observed in the availability of pharmaceuticals could also be due to (small) manufacturers not having the capacities to familiarise themselves with the various regulations [29–31], and co-licencing might not be an attractive option for smaller products in these markets. Moreover, manufacturers might not be interested in launching their products in markets with lower expected prices and smaller expected market size because the cost of doing so is disproportionately large compared to the expected turnover [32]. This is also in line with the results of our correlation analysis, which indicates that countries with a higher GDP have a shorter launch delay. Using countries with above-average availability and below-average launch delays as a benchmark, stakeholders may discuss or modify current pharmaceutical policy, if needed, to move towards shorter launch delays and greater market availability.

Prior research also suggests that price regulation delays launches [33–35], and because a low price in one market can spill over to other markets through parallel trading and external referencing, manufacturers may prefer a longer launch delay or no launch at all over a lower price [36]. On the other hand, health technology assessment institutions, as well as economic and demographic factors that make markets more profitable, can speed up diffusion [37].

It is widely accepted that accessible, quality pharmaceuticals can improve patients’ health substantially and enable patients to live longer and healthier lives. Thus, a high coverage of pharmaceuticals is needed across all countries. Based on EML findings, we saw that while launch delay results were more or less the same compared to all other new pharmaceuticals, results for availability were higher for EMLs. However, there were still differences between countries (e.g. Germany:100% EML availability in 2011–2014 vs. Bosnia: 53.8% EML availability in 2011–2014).

Looking now in closer detail at specific indication areas (subgroup analyses with diabetes and psoriasis) we see that every European country from our dataset had pharmaceuticals to treat these diseases on the market. For example, both Bosnia-Herzegovina and Serbia had an availability of 25.0% in these indication areas, while in some countries there was 100% availability for all compounds for both diseases (e.g. Germany, the UK, the Netherlands and Switzerland). Thus, it seems that even in countries with a small market size and low expected prices, drugs were available, even if not all of them.

Results suggest that countries with lower expected prices or smaller expected market size (i.e., smaller than the average) have a longer launch delay and a lower availability of pharmaceuticals, whereas countries with higher expected prices or larger expected market size (i.e., larger than the average) have a shorter launch delay and a higher availability of pharmaceuticals.

Future research could investigate whether there are variations in launch delay and the availability of pharmaceuticals across different groups of indications. We were not able to investigate this with our data set because it was not possible to filter pharmaceuticals by ATC Codes or indications. Developing a deeper, causal understanding of the pathways that have led to the reduction we observed in launch delays over time would also be important.

### Limitations

Our analyses have several limitations. First, IQVIA’s methods for collecting data on launch dates differs slightly among countries. For example, launch dates for Estonia, Greece, and Luxembourg are calculated based on reports from the retail sector, whereas launch dates for all other countries are calculated based on both the retail and hospital sectors. However, the potential bias this might have introduced to our measure of launch delay and the ranking order of launches is probably small because launch dates from the retail and hospital sectors do not differ significantly. Nevertheless, we had to exclude Estonia, Greece and Luxembourg three countries in our analysis of the availability of pharmaceuticals.

Second, our calculations of launch delay can be based only on available data. Especially in cases where new products have not yet gained market access in all countries in which a launch is planned, the launch delays could be longer than is suggested by the results of our analysis. This is particularly the case for the 4th time interval (2015–2017) in our observation period. However, except for Bosnia-Herzegovina, Bulgaria, Croatia, Estonia, France, Hungary, Lithuania, Serbia, and Turkey, the average launch delay in 2015 was already shorter than two years, minimizing the impact on our analysis of having only three years of follow-up for most countries in our sample.

Third, in some countries (e.g. Germany), a new drug can be accessed immediately at launch and is reimbursed for all indications by payers. However, this is not the case in every country. In some countries, only parts of the indication are reimbursed or reimbursement is restricted to specific groups. Thus, our measure of launch delay -based on launch dates and not actual use - have certain limits when it comes to evaluating patient access.

Lastly, two researchers independently reviewed all pharmaceuticals that were launched in fewer than five countries to verify some of the launch dates in the database. Although carried out to the best of our abilities, manual checks are always accompanied by uncertainties. However, because two reviewers independently performed the check, errors should have been reduced to a minimum.

## Conclusion

With this paper we provide an extensive overview of the availability of new pharmaceuticals and delays in their launch in 30 European markets over the past two decades. We found a clear trend towards a reduction in launch delays across all countries in our sample from 2000 (37.92 months) to 2017 (11.96 months). The three fastest launching countries were the Netherlands, Sweden, and Germany, and the slowest were Bosnia-Herzegovina, Serbia, and Turkey. Germany had the highest availability of new pharmaceuticals over the whole period (85.7%), followed by the UK (83.1%), and Norway (82.9%). GDP per capita was negatively correlated with launch delay (-0.67, *p* < 0.000) and positively associated with the availability of pharmaceuticals (+ 0.19, *p* < 0.000). This result suggests that countries with lower expected prices or smaller expected market size have fewer launches and longer launch delays, whereas countries with higher expected prices and larger expected market size have more launches and shorter launch delays.

Our findings can inform discussions about pharmaceutical policy and help policymakers pinpoint where existing regulations and laws may require improvement in order to achieve shorter launch delays and higher market availability for new pharmaceuticals. Countries with above average availability and below average launch delays may serve as a benchmark in this regard.

## Supplementary Information

Additional file 1: **Table S1.** Correlation analysis.

Additional file 2: **Table S2.** Launch delay for all pharmaceuticals vs. EMLs. 

## Data Availability

The data that support the findings of this study are available from IQVIA but restrictions apply to the availability of these data, which were used under license for the current study, and so are not publicly available. Data are however available from the authors upon reasonable request and with permission of IQVIA.
