# Supplementary material for: Changes in launch delay and availability of pharmaceuticals in 30 European markets over the past two decades
Source: BMC Health Serv Res. 2022 Nov 30;22:1457. doi: 10.1186/s12913-022-08866-7 (PMC9714155; doi:10.1186/s12913-022-08866-7)
Supplement: Supplementary file 1 — Table S1. Correlation analysis. [file 12913_2022_8866_MOESM1_ESM.pdf]

Table S1: Correlation analysis

	correlation coeff.	p-value
Launch delay and GDP	-0.67	<0.000
Availability and GDP	0.19	<0.000
