# Supplementary material for: Changes in launch delay and availability of pharmaceuticals in 30 European markets over the past two decades
Source: BMC Health Serv Res. 2022 Nov 30;22:1457. doi: 10.1186/s12913-022-08866-7 (PMC9714155; doi:10.1186/s12913-022-08866-7)
Supplement: Supplementary file 2 — Table S2. Launch delay for all pharmaceuticals vs. EMLs. [file 12913_2022_8866_MOESM2_ESM.pdf]

Table S2: Launch delay for all pharmaceuticals vs. EMLs

	Launch delay							
	**2000-2004**		**2005-2010**		**2011-2014**		**2015-2017**	
**Country**	**All N=3348**	**EMLs N=540**	**All N=3240**	**EMLs N=297**	**All N=4266**	**EMLs N=351**	**All N=2430**	***left out due to low N (=only 1 EML in this time period)**
Austria	23.07	25.30	21.02	18.16	13.06	6.63	8.34	
Belgium	34.60	32.54	25.00	24.65	24.24	13.21	18.33	
Bosnia	99.53	102.88	63.07	73.66	47.24	42.91	28.44	
Bulgaria	54.76	50.14	37.62	45.57	34.39	24.84	26.36	
Croatia	61.34	53.76	47.69	52.45	35.65	29.08	21.22	
Czech Republic	40.10	36.47	27.78	30.34	24.70	12.47	16.99	
Finland	22.86	19.84	18.49	19.30	15.65	9.53	11.61	
France	33.00	34.09	25.45	23.52	24.33	20.36	19.58	
Germany	19.44	20.11	14.40	18.56	10.93	5.92	7.49	
Hungary	39.62	30.87	27.14	27.41	26.68	11.97	20.38	
Ireland	19.63	23.12	23.74	19.27	18.81	10.89	18.04	
Italy	30.78	30.62	25.58	24.25	20.95	12.03	14.60	
Latvia	45.75	52.22	36.90	43.68	35.55	22.43	14.15	
Lithuania	48.36	50.47	46.05	72.26	33.53	26.52	21.23	
Netherlands	11.10	16.56	7.56	5.16	5.78	1.62	5.38	
Norway	26.69	21.89	22.05	19.30	15.11	11.71	10.41	
Poland	40.80	36.19	24.73	14.98	21.39	9.10	14.70	
Portugal	33.36	33.27	24.78	21.36	18.16	6.23	12.56	
Romania	51.90	47.16	41.16	41.92	31.42	20.95	21.89	
Serbia	71.90	62.78	59.47	73.57	46.21	41.68	32.57	
Slovakia	43.41	37.03	27.90	27.35	24.06	16.66	20.62	
Slovenia	44.89	45.02	31.72	29.73	24.28	15.73	16.56	
Spain	26.44	25.11	23.41	21.66	22.56	15.30	17.90	
Sweden	14.73	16.78	12.88	21.56	6.82	4.17	4.55	
Switzerland	26.13	18.84	29.78	20.78	19.88	7.49	15.62	
Turkey	46.76	40.63	50.88	50.89	40.63	22.84	29.02	
UK	20.42	22.63	17.00	16.77	11.23	4.30	7.52	
